# Rare Plankton Subcommunities Are Far More Affected by DNA Extraction Kits Than Abundant Plankton

**DOI:** 10.3389/fmicb.2019.00454

**Published:** 2019-03-11

**Authors:** Min Liu, Yuanyuan Xue, Jun Yang

**Affiliations:** ^1^Aquatic EcoHealth Group, Key Laboratory of Urban Environment and Health, Institute of Urban Environment, Chinese Academy of Sciences, Xiamen, China; ^2^University of Chinese Academy of Sciences, Beijing, China

**Keywords:** DNA extraction, high-throughput sequencing, plankton, species diversity, community composition

## Abstract

Advances in high-throughput sequencing technologies allow a more complete study of microbial plankton community composition and diversity, especially in the rare microbial biosphere. The DNA extraction of plankton is a key step for such studies; however, little is known about its influences on the abundant or rare microbial biosphere. Our aim was to quantify the influences of different DNA extraction kits on abundant and rare plankton in the surface waters of a reservoir and provide a reference for the comparisons between microbial community studies with different extraction methods. We evaluated the influence of five common commercial kits on DNA quality, microbial community diversity and composition, and the reproducibility of methods using both 16S and 18S rRNA genes amplicon sequencing. Our data showed that results of Fast DNA Spin Kit for Soil (MPF) had higher α diversity for bacteria and high DNA quality, indicating that it is the most suitable approach for bacterioplankton diversity study. However, DNeasy Blood & Tissue Kit (QD) and QIAamp DNA Mini Kit (QQ) methods could produce results that are easier to replicate for bacteria and eukaryotes, respectively, and were more comparable between studies. The use of different DNA extraction kits had larger influence on the rare taxa compared with abundant taxa. Therefore, the comparability between studies that employed different extraction methods can be improved by removing low-abundance or less-representative OTUs. Collectively, this study provides a comprehensive assessment of the biases associated with DNA extraction for plankton communities from a freshwater reservoir. Our results may guide researchers in experimental design choices for DNA-based ecological studies in aquatic ecosystem.

## Introduction

Recently, with the rapid development of sequencing technology DNA-based experiments have become routinely used to study the microbial community in various ecosystems (Shendure et al., 2017; Sinha et al., 2017; Knight et al., 2018). Microorganisms in aquatic ecosystems (i.e., oceans, rivers, lakes and reservoirs) which are some of the most studied ecosystems for their high microbial diversity, play key roles in biogeochemical processes (Liu et al., 2015; Sunagawa et al., 2015; Xue et al., 2018). Numerous studies have shown DNA-based approaches may provide unprecedented insight into the dynamics of microbial communities, and reveal general principles about their ecology and mechanisms of community assembly. However, several technical limitations are still underexplored in acquiring the DNA from water samples and constitute a key constraint to the accuracy of the new findings (Deiner et al., 2015; Walden et al., 2017).

Generally, different DNA extraction methods are recommended based on the origin of the samples as there is not a single superior method which can be applied across all samples and microbes (Kuhn et al., 2017; Sinha et al., 2017; Walden et al., 2017). Studies have showed suitable extraction methods for specific samples such as human fecal (Costea et al., 2017), soil protist (Santos et al., 2015, 2017) and fish (Eichmiller et al., 2016). The variable structure of microbial cell walls can lead to a misrepresentation of specific microbial taxa, increasing the difficulty of data comparison between studies (Cabeen and Jacobs-Wagner, 2005; Santos et al., 2017). So far, few researches have evaluated the influences of different extraction methods on bacterial and eukaryotic plankton communities, respectively (Eland et al., 2012; Albertsen et al., 2015; Li et al., 2015; McCarthy et al., 2015; Walden et al., 2017). Further, we have very limited knowledge how DNA extraction methods influence the plankton community of bacteria and eukaryotes together, with an emphasis on the comparison between abundant and rare plankton.

An important aspect that should be considered is the influence of DNA extraction methods on the rare biosphere of the microbial community. The study of these rare taxa has become a very active research area, due to the development of fast and cheap high-throughput sequencing and their important and unknown ecological roles (Sogin et al., 2006; Pedrós-Alió, 2012; Liu et al., 2015, 2017, 2019; Lynch and Neufeld, 2015; Xue et al., 2018). However, until now, only a few studies have evaluated the influences of DNA extraction kits on rare taxa (Supplementary Table S1). Limited studies have showed that kits contamination of different methods may be different and would have a high influence on microbial samples with low biomass (Salter et al., 2014; Velásquez-Mejía et al., 2018). To better compare results collected from different research groups or studies, the evaluation of the influence of DNA extraction methods on taxa in different relative abundances is an urgent call.

The reproducibility of each DNA extraction method is another important aspect which should be considered and assessed. The reproducibility has a very important role in comparing data from different studies, labs, or even within the same study (Zhou et al., 2011; Sinha et al., 2017). Here we collected one surface water sample from a subtropical reservoir, and systematically tested how distinct DNA extraction methods influence on the taxa in different relative abundances (i.e., rare and abundant taxa), instead of the effect of downstream analysis (i.e., PCR, primer choice, DNA sequencing platform, and bioinformatics). Additionally, sequencing depth was also considered to better estimate the reproducibility of each DNA extraction method.

The aim of this study was to inform the experimental design by quantifying the relative influence of DNA extraction on plankton taxa with different relative abundances, and to provide a reference for the comparison of different studies on microbial plankton communities, instead of obtain the “best” protocols for aquatic microbiome studies. We compared five commonly used DNA extraction kits, using the 16S rRNA and 18S rRNA gene amplicon sequences data as the readout, and evaluated taxonomic variability of both bacterial and microeukaryotic plankton. Specifically, we assessed: (i) the influence of DNA extractions on the bacterial and eukaryotic diversity; (ii) the influence of DNA extraction kits on plankton community composition based on different distances; (iii) the similarity of community composition detected from the replicate extractions, which indicates the reproducibility of each method.

## Materials and Methods

### Experimental Design

Water samples (upper 50 cm, 15 litter) were collected by a 5-L polymethyl methacrylate sampler in Tingxi Reservoir (24°48′N, 118°08′E) on July 26, 2016. Then all waters were put into a 20-L PVC bottle and transferred to the laboratory as soon as possible. The samples were first filtered through a 200 μm pore-size sieve to remove debris, large metazoans and grains. Then plankton communities (500 mL water) were collected on each 0.22 μm pore-size polycarbonate filter (47 mm diameter, Millipore, Billerica, MA, United States). The water was well mixed in the 20-L PVC bottle before filtering to keep the uniformity of each filtered sample. The filters were then stored at −80°C until DNA extraction.

Five commercial DNA extraction kits from three companies which are commonly used to extract DNA from environmental samples (Lear et al., 2018), were evaluated in this study (Table 1). Commercial DNA extraction kits were used in this study for their standardized application in multiple-labs (Renshaw et al., 2015; Walden et al., 2017). The DNA extractions were carried out in triplicate for each kit, and subsequent analyses were performed on the 15 individual extracts. All triplicates were taken from the same water and same bottle (20-L PVC bottle). Negative control extractions, where new membranes without plankton samples were added, were also performed for each method in triplicate. Each membrane or filter represents one replication. We mostly followed the instructions of manufacturers but introduced changes to improve the comparability among different DNA extraction kits. For MBS kit, to make it comparable to other DNA extraction kits, instead of filtering by Sterivex^TM^ filter unit, the filtered membrane was cut into small pieces and put into a sterilization centrifuge tube. Then we added the 0.9 mL of solution ST1B, vortexed as instruction and followed by adding 0.9 mL of ST2. After incubation at 90°C for 5 min, the mixtures were cooled at room temperature for 2 min. Next, the mixtures were vortexed at maximum speed for 5 min. After this, the lysate was added into the 5 mL PowerWater^®^ Sterivex^TM^ glass Bead Tube. The other steps followed the manufacturer instructions to extract the DNA. The extracted DNA was quantified by a NanoDrop 1000 spectrophotometer (Thermo Fisher Scientific, Pittsburgh, PA, United States) and stored at −20°C until further use. Extracts were considered to contain sufficiently pure genomic DNA when their A260/A280 nm ratio was between 1.8 and 2.0. Otherwise, the extracted DNA included proteins, phenols or other contaminants (Hermans et al., 2018).

**Table 1:** The five common DNA extraction kits used in this study.

Extraction kits	Abbreviation	Lysis method	Company	Headquarter address
PowerWater DNA isolation kit	MB	Mechanical, chemical	MoBio Laboratories Inc.	Carlsbad, CA, United States
PowerWater^®^ Sterivex^TM^ DNA isolation kit	MBS	Mechanical, chemical	MoBio Laboratories Inc.	Carlsbad, CA, United States
Fast DNA spin kit for soil	MPF	Mechanical, chemical	MP Biomedicals	Santa Ana, CA, United States
DNeasy blood and tissue kit	QD	Heat, chemical, enzymatic	Qiagen	Hilden, Germany
QIAamp DNA mini kit	QQ	Heat, chemical	Qiagen	Hilden, Germany

### PCR Amplification and Illumina Sequencing

We used primer pairs targeting the V3-V4 variable region of 16S rRNA gene in bacteria (341F, 5′-CCTAYGGGRBGCASCAG-3′; 806R, 5′-GGACTACNNGGGTATCTAAT-3′) (Yu et al., 2005; Sundberg et al., 2013; Guo et al., 2018) and the V9 variable region of the 18S rRNA gene in eukaryotes (1380F, 5′-CCCTGCCHTTTGTACACAC-3′; 1510R, 5′-CCTTCYGCAGGTTCACCTAC-3′) (Amaral-Zettler et al., 2009; Liu et al., 2017; Xue et al., 2018). Each DNA sample and negative control were run in triplicate. Each DNA sample was individually PCR-amplified in 30 μL reactions included an initial denaturation at 98°C for 1 min, followed by 30 cycles of 10 s at 98°C, 30 s at 50°C, and 30 s at 72°C. At the end of the amplification, the amplicons were subjected to final 5 min extension at 72°C. The 30 μL PCR mixture included 15 μL of Phusion^®^ High-Fidelity PCR Master Mix (New England Biolabs, Beverly, MA, United States); 0.2 μM of forward and reverse primers, and about 10 ng template DNA for both bacteria and eukaryotes. The triplicate PCR products were mixed in equimolar amounts and were confirmed after running in 1% agarose gel. Then the PCR products were isolated from the gel and purified with GeneJET Gel Extraction Kit (Thermo Fisher Scientific, Hudson, NH, United States). Sequencing libraries were constructed using NEB Next Ultra DNA Library Prep Kit for Illumina (New England Biolabs, Ipswich, MA, United States) following manufacturer’s instructions, and barcodes were added. The library quality was estimated on the Qubit 2.0 Fluorometer (Thermo Fisher Scientific, Waltham, MA, United States) and Agilent Bioanalyzer 2100 system (Agilent Technologies, Palo Alto, CA, United States). At last, the library was sequenced on the Illumina MiSeq platform (Illumina, Inc., San Diego, CA, United States) using a 250 bp paired-end protocol (Liu et al., 2017).

### Bioinformatics

Paired-end reads were merged by using FLASH (Magoč and Salzberg, 2011) and then assigned to each sample according to the unique barcodes. Sequence data were processed using quantitative insights into microbial ecology (QIIME v.1.8.0) software following standard protocols: maximum number of consecutive low-quality base = 3; minimum of continuous high-quality base = 75% of total read length; maximum number of ambiguous bases = 0 (Caporaso et al., 2010). Chimeric sequences were identified by UCHIME and discarded before further analysis (Edgar et al., 2011). Quality-filtered sequences were then assigned to operational taxonomic units (OTUs) at 97% level of sequence similarity by using the pick_otus.py. The 97% threshold has been widely used for both of bacteria and eukaryotes (Henderson et al., 2013; Liu et al., 2015, 2017; Dai et al., 2016; Hermans et al., 2018; Xue et al., 2018). There is no general agreement on a standard definition to classify the eukaryotes into OTUs at species level. We selected 97% threshold for eukaryotic plankton to facilitate comparisons between studies because many previous studies used this threshold to define OTUs (Liu et al., 2017; Xue et al., 2018). Further, our previous study (Liu et al., 2017) found that the choice of different thresholds (97% vs. 99%) had no apparent effect on overall results and general conclusions in plankton community ecology. Sequences were taxonomically classified by the RDP classifier using the 80% confidence threshold against the Silva 123 for bacteria (Quast et al., 2013) and PR^2^ for eukaryotes (Guillou et al., 2013), respectively. For bacteria, all eukaryota, chloroplasts, archaea, mitochondria, unknown sequences and singleton OTUs were excluded. For eukaryotes, unassigned and singleton OTUs were excluded. Finally, sequences data were normalized to 41,744 and 121,146 sequences per sample using the “sub.sample” command in MOTHUR v.1.33.3 (Schloss et al., 2009) for bacteria and eukaryotes, respectively.

All raw sequences from this study have been submitted to the National Center for Biotechnology Information (NCBI) Sequence Read Archive (SRA) database under the BioProject number PRJNA474064 and the accession number SRP149868.

### Definition of Rare and Abundant Plankton

In order to evaluate the effects of DNA extraction kits on taxa in different relative abundances, we expanded the classification of microbial taxa based on the detected sequences and their relative abundance. This followed the definition of abundant (1%) and rare (0.01%) biosphere in previous studies (Pedrós-Alió, 2012; Liu et al., 2015; Xue et al., 2018). All OTUs were artificially defined and grouped into 6 exclusive categories following previous studies (Dai et al., 2016; Xue et al., 2018; Liu et al., 2019): (i) the OTUs with a relative abundance always ≥1% in all replicates were regarded as always abundant taxa (AT); (ii) the OTUs with a relative abundance greater than 0.01% in all replicates and ≥1% in some replicates but never rare (<0.01%) were regarded as conditionally abundant taxa (CAT); (iii) the OTUs with a relative abundance varying from rare (<0.01%) to abundant (≥1%) were regarded as conditionally rare and abundant taxa (CRAT); iv) the OTUs with relative abundance between 0.01% and 1% in all replicates were regarded as moderate taxa (MT); v) the OTUs with a relative abundance <0.01% in some replicates but never ≥1% in all replicates were regarded as conditionally rare taxa (CRT); (vi) the OTUs with a relative abundance always <0.01% in all replicates were regarded as always rare taxa (RT).

### Data Analyses

Rarefaction curves, and α-diversity indices were computed by MOTHUR v.1.33.3 (Schloss et al., 2009). The non-parametric Kruskal-Wallis test was used to evaluate the influences of DNA extraction kits on α-diversity indices and the quality and quantity of DNA by SPSS 22.0 (IBM Corp., Armonk, NY, United States).

Four dissimilarity matrices (Bray-Curtis, Jaccard, weighted unifrac and unweighted unifrac) within and between DNA extraction kits were calculated with the relative abundance-based OTUs of bacteria and eukaryotes. Analysis of similarities (ANOSIM) was used to estimate the significant differences among different DNA extraction kits. Complete separation is suggested by *R* = 1, with *R* = 0 representing no separation (Clarke and Gorley, 2015). We used the adonis function (vegan R packages) to run a PERMANOVA on the Bray-Curtis dissimilarity profiles using 10,000 permutations for assessing the effect of DNA extraction methods (Anderson, 2001). Similarity of percentages analysis (SIMPER) analysis was performed with PAST (Paleontological Statistics, version 3.01) software to identify the contribution of each OTU to the community dissimilarity (Hammer et al., 2001). The Bray-Curtis dissimilarity was used as the distance for ANOSIM, PERMANOVA and SIMPER analyses.

The reproducibility of each DNA extraction kit, evaluating the difference between plankton community compositions among triplicates within each DNA kit, was quantified by computing the average dissimilarity of each set of three replicates, using four dissimilarity matrices. The lower the dissimilarity measure, the more consistent that method was predicted to be. In addition, we randomly selected subsets of cleaned sequences (10,000, 20,000, 30,000, and 40,000 for bacteria; 10,000, 20,000, 30,000, 40,000, 50,000, 60,000, 70,000, 80,000, 90,000, and 100,000 for eukaryotes) from each replicate to estimate the influence of sequencing depth on the reproducibility. The one-way ANOVA was used to test the significant difference for reproducibility of DNA extraction kits.

A Venn diagram was constructed using the “Venn Diagram” package to compare the number of OTUs detected by using different DNA extraction kits. The “Niche breadth” approach of Levins (1968) was used to evaluate DNA extraction kits preference by the formula:

(1) $$B_{j}=\frac{1}{\sum_{i=1}^{N}P_{\mathrm{ij}}^{2}}$$

where *B*_j_ indicates the number of DNA extraction kits which the OTU occurred and *P*_ij_ represents the percentage of individuals belonging to species j present in a given DNA extraction kit *i*. Phylotypes characteristic of specific DNA extraction kit were identified using indicator species analysis (ISA). Those indicator OTUs with a *P*-value <0.05 and phylotypes with indicator values >50, were considered valid (Dufrene and Legendre, 1997). A heat map of the 20 most abundant OTUs and high-rank taxa was made using the “pheatmap” package in the R environment.

## Results

### Effect of Extraction Kits on Plankton DNA Quality and Quantity

Although the DNA purity of the same filtered samples had no significant difference across different DNA extraction kits, the DNA concentrations varied greatly (Supplementary Figure S1). The DNA purity ranged from 1.90 to 2.15. The best results were obtained using PowerWater DNA Isolation Kit (MB) (mean, 1.97), PowerWater^®^ Sterivex^TM^ DNA Isolation Kit (MBS) (1.95) and Fast DNA Spin Kit for soil (MPF) (1.93). The highest DNA concentration was obtained using Fast DNA Spin Kit for soil (MPF) (mean ± s.e., 64.80 ± 1.12 ng/μL), and PowerWater DNA Isolation Kit (MB) (15.85 ± 2.90 ng/μL) produced the lowest yield of DNA. Overall, MPF can maximize DNA concentration and purity. All negative extraction controls yielded DNA below the limit of detection.

### Effect of Extraction Kits on Plankton Richness and α-Diversity

**FIGURE 1:**
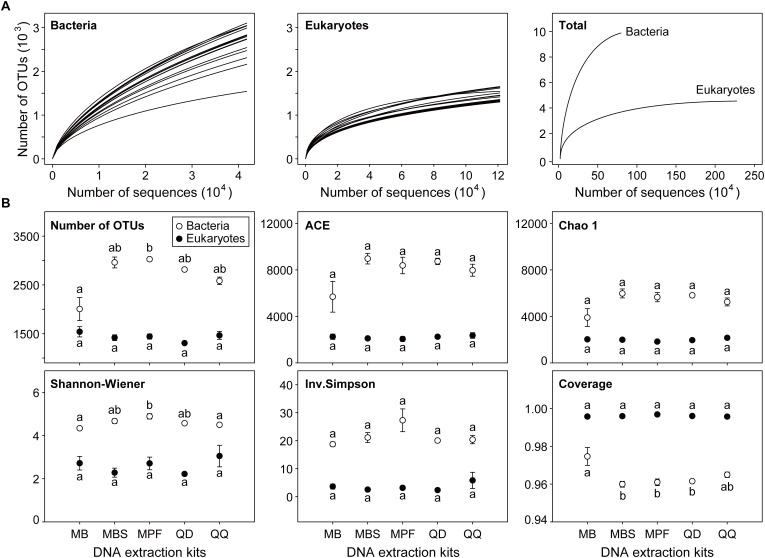
The effect of DNA extraction kits on α-diversity of both bacterial and eukaryotic plankton. **(A)** Rarefaction curves of each single community and the combined communities for bacteria and eukaryotes, respectively. **(B)** Comparison of community diversity parameters. The OTUs were defined at 97% sequence similarity level. All data are means ± standard error (*n* = 3). Significant difference (*P* < 0.05) is indicated by different letters of the alphabet, and the significance is calculated by non-parametric Kruskal-Wallis test.

Most of the microbial plankton taxa had been recovered, as indicated by the species accumulation curves (Figure 1A), although no single replicate sample achieved a full saturation in the rarefaction curves. The sequencing depth for eukaryotic plankton (121,146) was higher than that of bacteria (41,744). Distinct influences of DNA extraction kits on α-diversity indices were observed for bacteria and eukaryotes (Figure 1B). For bacteria, only observed OTU number and Shannon-Wiener index had significant differences among DNA extraction kits (*P* < 0.05). MB showed the lowest OTU number (2005 ± 235) and Shannon-Wiener (4.34 ± 0.02), however the highest values of OTU number (3027 ± 19) and Shannon-Wiener (4.89 ± 0.12) were obtained from MPF. For eukaryotes, no significant difference was found for any α**-**diversity index among five different DNA extraction kits.

### Reproducibility and Comparability of DNA Extraction Kits

To evaluate the effects of extraction kits on rare and abundant microbial taxa, we defined and grouped all OTUs into six exclusive categories based on their ranges of the relative abundance in 15 replicate samples (Supplementary Figure S2). Rare taxa had the highest OTUs number for both bacteria (86.62%) and eukaryotes (80.75%), whereas abundant taxa had the lowest OTUs number for both bacteria (0.04%) and eukaryotes (0%). For bacteria, conditionally abundant taxa showed the highest sequence number (42.65%), while the lowest sequence number was found in conditionally rare and abundant taxa (1.32%). For eukaryotes, conditionally abundant taxa also showed the highest sequence number (57.71%), while the lowest sequence number was found in abundant taxa (0%).

**FIGURE 2:**
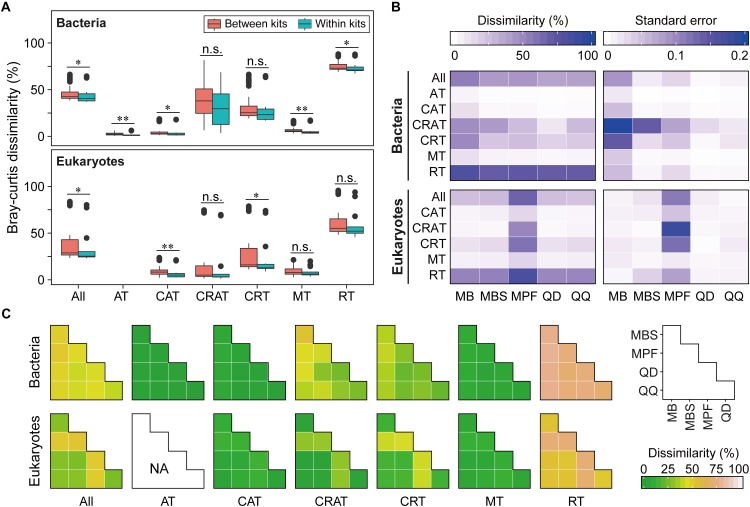
The effect of DNA extraction kits on community composition of both bacterial and eukaryotic plankton based on Bray-Curtis dissimilarity. **(A)** The overview of pairwise Bray-Curtis dissimilarity of bacterial and eukaryotic plankton communities based on five different DNA extraction kits, respectively. Statistical analysis is non-parametric Mann-Whitney *U* test. ^∗^*P* < 0.05, ^∗∗^*P* < 0.01, n.s. *P* > 0.05. **(B)** Variation in the community composition of six OTUs categories produced by three replicates within the DNA extractions kits. The data were expressed as mean Bray-Curtis dissimilarity (left) and its standard error (right), with a more dark blue indicating a more dissimilar or larger variation. **(C)** Pairwise Bray-Curtis dissimilarity of bacterial and eukaryotic plankton communities between different DNA extraction kits. Note that no always abundant taxa (AT) was identified for eukaryotic plankton in this study. AT, always abundant taxa; CAT, conditionally abundant taxa; CRAT, conditionally rare and abundant taxa; CRT, conditionally rare taxa; MT, moderate taxa; RT, always rare taxa.

Community dissimilarity can be mainly due to the differences of DNA extraction methods (Figure 2A and Supplementary Figures S3A–S5A). The reproducibility of each DNA extraction kit showed significant difference (*P* < 0.05). QD and QQ exhibited the highest consistency for bacteria (0.62) and eukaryotes (0.76), respectively (Figure 2B). Different DNA extractions showed distinct patterns of consistency along relative abundance ranks (Figure 2 and Supplementary Figures S3–S5). Interestingly, RT, CRT and CRAT had lower consistency compared with AT, CAT and MT categories for both bacteria and eukaryotes (Figure 2B), indicating that DNA extractions have the larger effects on taxa with the lower relative abundance. In general, the higher the average between-replicate dissimilarity was, the larger the standard error of the dissimilarity was estimated (Figure 2 and Supplementary Figures S3–S5). Furthermore, we did not find that the sequencing depth showed any significant influence on the reproducibility of each DNA extraction kit for eukaryotes from 10,000 to 100,000 sequences (Supplementary Figure S6). For bacterial community, however, the reproducibility of both QD and QQ was significant higher for 40,000 sequencing depth than 10,000 sequencing depth (*P* < 0.05), while sequencing depth exhibited no significant effect on the reproducibility of three other DNA extraction kits from 10,000 to 40,000 sequences (Supplementary Figure S6).

### Effects of Extraction Kits on Community Composition

Four kinds of distance indices were considered to compare the community differences among the DNA extraction kits based on the variation of OTUs along relative abundance ranks, and in general they produced similar results (Figure 2C and Supplementary Figures S3C–S5C). For both bacteria and eukaryotes, most of the community differences were from rare taxa. By contrast, abundant, conditionally abundant, and moderate taxa showed higher similarity and higher consistency among different DNA extraction kits. In addition, two non-parametric multivariate statistical tests (ANOSIM and Adonis) showed that these observed differences were statistically significant (Table 2). Furthermore, the SIMPER analysis showed that RT and CRT accounted for the largest contribution for community differences for both bacteria (69.62% for RT, 27.86% for CRT) and eukaryotes (59.56% for RT, 38.24% for CRT) (Supplementary Figure S7).

**Table 2:** Analysis of similarity (ANOSIM) and permutational multivariate analysis of variance (PERMANOVA) for comparisons of microbial plankton communities among five different DNA extraction kits based on Bray-Curtis dissimilarity.

	ANOSIM				PERMANOVA			
Category	Bacteria		Eukaryotes		Bacteria		Eukaryotes	
	R	*P*-value	R	*P*-value	R^2^	*P*-value	R^2^	*P*-value
All	**0.34**	**0.004**	**0.32**	**0.001**	**0.33**	**0.006**	**0.37**	**0.002**
AT	**0.48**	**0.001**	–	–	**0.50**	**0.009**	–	–
CAT	**0.40**	**0.001**	**0.51**	**0.001**	0.30	0.318	**0.54**	**0.001**
CRAT	**0.26**	**0.008**	**0.32**	**0.002**	0.40	0.073	**0.37**	**0.002**
CRT	**0.28**	**0.005**	**0.33**	**0.003**	0.33	0.150	**0.38**	**0.005**
MT	**0.58**	**0.001**	**0.18**	**0.040**	**0.39**	**0.016**	0.35	0.241
RT	**0.35**	**0.001**	**0.26**	**0.002**	**0.31**	**0.003**	**0.34**	**0.002**

*Bold font indicates the significance at P < 0.05. Values show the R and R^2^ values for ANOSIM and PERMANOVA, respectively. The operational taxonomic units (OTUs) were defined at 97% sequence similarity threshold. The ANOSIM statistic compares the mean of ranked dissimilarities among groups to the mean of ranked dissimilarities within groups. An R value close to “1” suggests dissimilarity between groups, while an R value near “0” suggests an even distribution of high and low ranks within and between groups. Negative R values indicate that dissimilarities are greater within groups than between groups. AT, always abundant taxa; CAT, conditionally abundant taxa; CRAT, conditionally rare and abundant taxa; CRT, conditionally rare taxa; MT, moderate taxa; RT, always rare taxa. Note that no always abundant taxa (AT) was identified for eukaryotic plankton in this study.*

**FIGURE 3:**
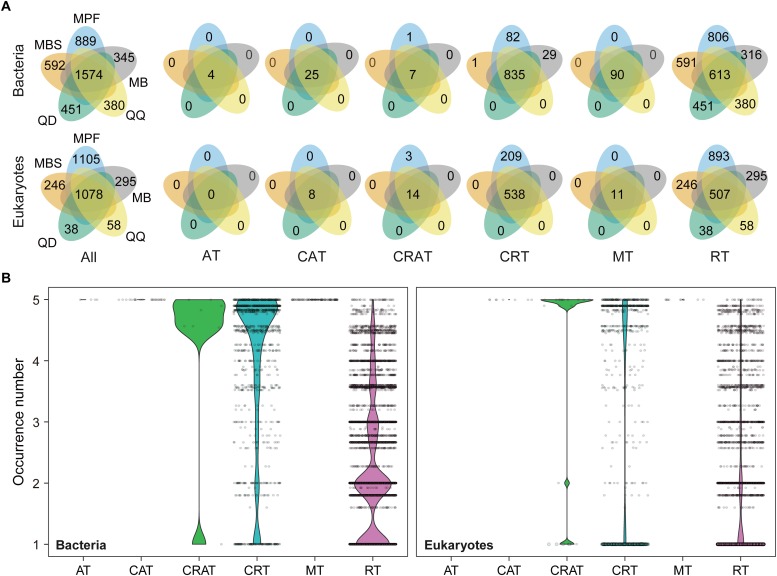
The effect of DNA extraction kits on the OTUs occurrence. **(A)** Venn diagram showing the number of OTUs that shared and unique by different DNA extraction kits. **(B)** The occurrence (that is, the number of DNA extraction kits where an OTU was found) based on niche breadth analysis for all the bacterial and eukaryotic OTUs, respectively. AT, always abundant taxa; CAT, conditionally abundant taxa; CRAT, conditionally rare and abundant taxa; CRT, conditionally rare taxa; MT, moderate taxa; RT, always rare taxa.

**FIGURE 4:**
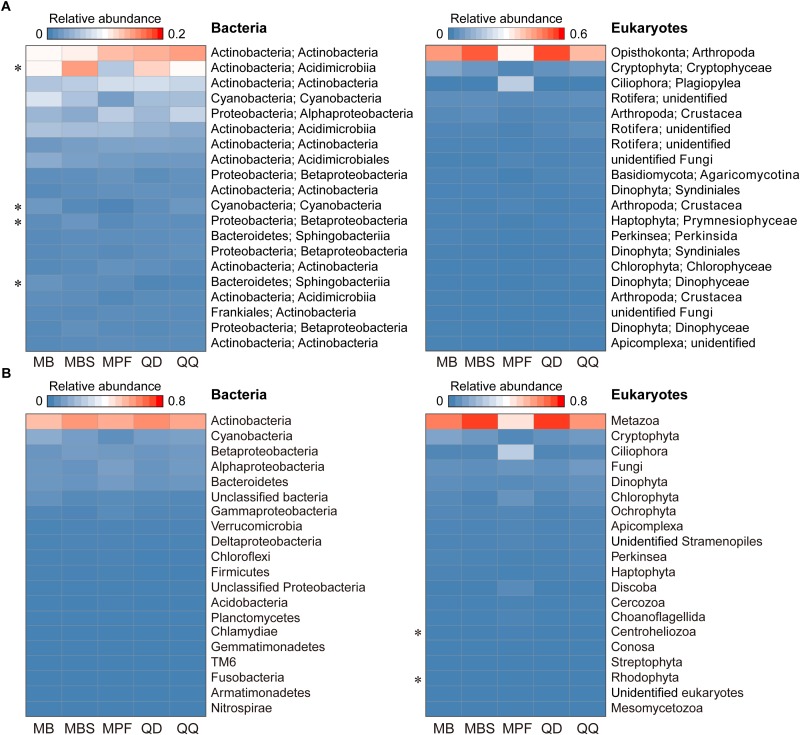
The effect of DNA extraction kits on the community composition. **(A)** Heat map of 20 most abundant OTUs in bacterial and eukaryotic plankton dataset. **(B)** Variation of main high-rank taxa of bacterial and eukaryotic plankton within each of DNA extraction kit. Statistical analysis is non-parametric Kruskal-Wallis test. ^∗^*P* < 0.05. Each OTU or high-rank taxon is in a row, and color intensity indicates its average relative abundance of each DNA extraction kit (*n* = 3).

The influence of DNA extraction kits on community composition was identified at both OTU and higher taxonomic levels (Figure 3, 4). At OTU level, significant differences were found for specific taxa. First, regardless the microbial plankton types, most of the unique OTUs for each method belonged to the rare taxa (Figure 3A). In total, 15% bacterial OTUs and 23% eukaryotic OTUs were identified in at least four extraction kits, and less than 60% OTUs were found in no more than two methods for both bacteria and eukaryotes (Figure 3B). Second, indicator OTUs were identified for each DNA extraction kit (Supplementary Table S2). For bacteria, the numbers of indicator OTUs among kits were 1 for MB, 0 for MBS, 6 for MPF, 0 for QD, and 2 for QQ methods. For eukaryotes, the numbers of indicator OTUs were 4 for MB, 0 for MBS, 13 for MPF, 0 for QD, and 7 for QQ methods. At last, only 4 (Actinobacteria, Cyanobacteria, Proteobacteria, Bacteroidetes) of 20 most abundant OTUs were significantly different among DNA extraction kits for bacteria, whereas no difference was identified for eukaryotic plankton (Figure 4A). At high-rank taxa level, no any significant difference was found for bacteria, while both Rhodophyta and Centroheliozoa were significantly different among five DNA extraction kits for eukaryotes (Figure 4B).

**FIGURE 5:**
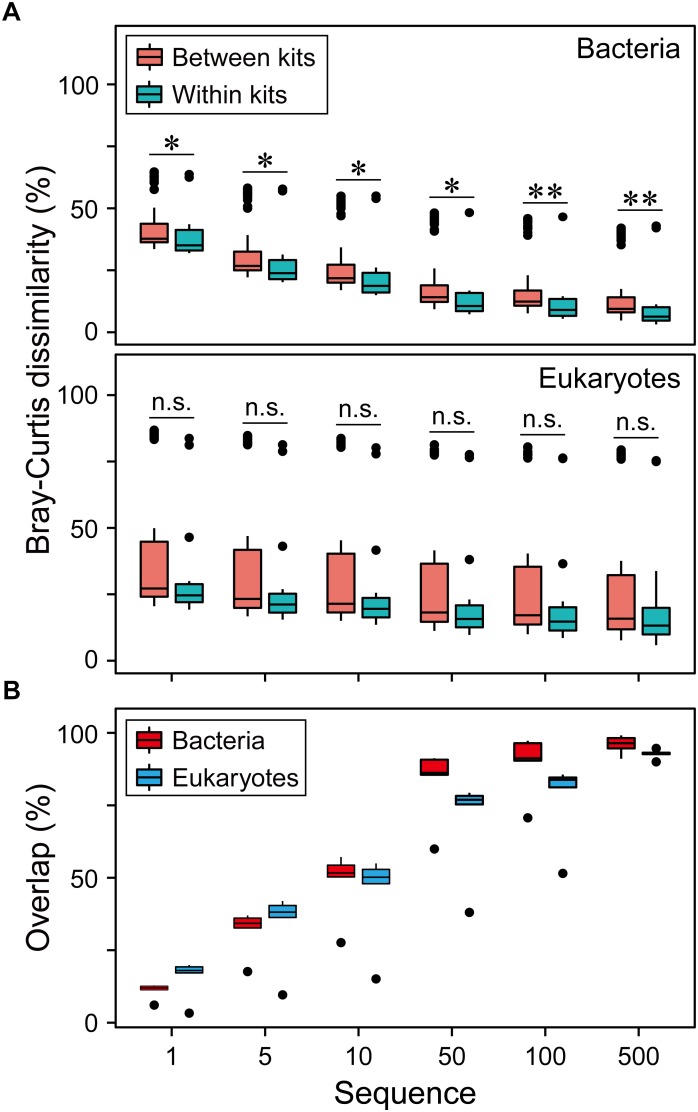
Effects of data preprocessing on resolving the differences of microbial communities. **(A)** The overview of pairwise Bray-Curtis dissimilarity of bacterial and eukaryotic plankton communities based on five different DNA extraction kits after the OTUs with < = 1, 5, 10, 50, 100, 500 sequences were removed. For bacteria, linear regression resulted in a significant pattern for between kits (*R*^2^ = 0.19, *P* < 0.01) and within kits (*R*^2^ = 0.14, *P* < 0.01). For eukaryotes, linear regression resulted in a significant pattern only for between kits (*R*^2^ = 0.01, *P* < 0.01). **(B)** The overlap of three replicates of bacterial and eukaryotic plankton communities based on five different DNA extraction kits after the OTUs with < = 1, 5, 10, 50, 100, 500 sequences were removed. Linear regression resulted in a significant correlation for bacteria (*R*^2^ = 0.37, *P* < 0.01) and eukaryotes (*R*^2^ = 0.45, *P* < 0.01).

Finally, to improve data comparability, we removed singleton sequences and OTUs with less and equal to 5, 10, 50, 100, 500 sequences. We found that the larger number of sequences removed, the lower the community dissimilarity (Figure 5A) and the higher overlap of the OTUs (Figure 5B) for both bacteria and eukaryotes. This indicates that the low-abundance taxa had significant contribution to the community dissimilarity.

## Discussion

With the development of high-throughput technologies of target genes, researchers can now directly quantify the rare biosphere, which play very important ecological and biogeochemical roles in various ecosystems (Sogin et al., 2006; Pedrós-Alió, 2012; Liu et al., 2015, 2019; Lynch and Neufeld, 2015; Xue et al., 2018). However, great caution is needed when using DNA-based sequencing technologies for describing and characterizing the diversity and composition of microbial community. The aim of this study is to comprehensively and systematically understand how low-abundance taxa were influenced by different DNA extraction kits. Here, we compared the variations of plankton communities from a single water sample using five of the most commonly used DNA extraction kits. We found that different DNA extraction kits had distinct influences on the alpha-diversity, community composition and specific taxa for both bacteria and eukaryotes. Specifically, low-abundance taxa were greatly affected by different DNA extraction kits. We also found that the community composition reproducibility varied among the DNA extraction kits.

The alpha-diversity of a microbial community could be influenced by DNA extraction kits to different degrees. Previous studies have found that the choice of DNA extraction methods could affect the alpha-diversity of bacterial communities from kick-net, leaf litter, soil and water (Purswani et al., 2011; Deiner et al., 2015; Hermans et al., 2018). However, no significant difference in alpha-diversity was obtained for eukaryotic (animal, fungal and plant) communities from soil and water samples (Hermans et al., 2018). Another study showed that no significant differences were found among DNA extraction kits when assessing diversity of eukaryotic microalgae from freshwater by using denaturant gradient gel electrophoresis (Eland et al., 2012). Compared with the previous studies mentioned above, sequencing depths were higher in this study. Our results indicated that the alpha-diversity of bacteria was significantly affected by different DNA extraction kits, whereas there was no significant influence on the alpha-diversity of eukaryotic plankton community.

Additionally, significant differences were found for specific taxa at OTU or phylum level for both bacteria and eukaryotes. Previous studies have shown the preference in extraction specific taxa such as cyanobacteria, Excavata, Cercozoa and Amoebozoa (Singh et al., 2011; Santos et al., 2017). The differences in community composition detected may partly be due to the distinct susceptibility of bacterial and eukaryotic cell wall to different lysis methods or beads of different materials and sizes (Bürgmann et al., 2001; Fredricks et al., 2005; Henderson et al., 2013; Velásquez-Mejía et al., 2018). Mechanical lysis is suitable for bacteria attributed to its harder lysing cells, whereas direct lysis of cells based on bead-beating protocols is considered to be more suited to the detection of microeukaryotes, as these cells often have fortified cell walls or very resilient cell membranes (Santos et al., 2017; Hermans et al., 2018). Otherwise, even for bacteria, methods without a bead beating or enzymatic treatment step generally extracted less DNA from Gram-positive bacteria (Knudsen et al., 2016). Other factors such as sample type or origin (Knudsen et al., 2016), inherent specimen properties (Knudsen et al., 2016; Sinha et al., 2017), primer choice (Albertsen et al., 2015) and bioinformatics protocol choices (Sinha et al., 2017) can also influence the outcome of microbial community analysis. It is difficult to find a single extraction method for all circumstances. Our results highlighted that it is important to design specific DNA extraction method to target particular taxonomic groups.

Previous studies have found the influence of DNA extraction kits on the whole or abundant taxa in microbial communities (Knudsen et al., 2016; Sinha et al., 2017; Hermans et al., 2018), however, the influence of extraction methods on low-abundance taxa were not comprehensively and systematically evaluated. Recently, Weiss et al. (2014) found that DNA extraction kits had significant influence on the resulting microbial community profile of low-biomass samples. Our results indicated that low-abundance taxa were largely influenced by the DNA extraction kits, and offered further evidence supporting that plankton taxa in different relative abundances were disproportionately influenced. One explanation for the results is that as the number of “true” taxa becomes less, the potential for contaminants occupying a larger fraction of the sequences will become greater (Weiss et al., 2014). Another explanation for this is that some of the community differences between DNA extraction kits may be actually “real” chance differences between replicate samples. It’s possible that some replicate samples may not contain any individuals of a very rare taxon, despite the fact that we mixed the water before filtering. In addition, the differences in microbial communities may also come from the amplicon sequencing-based detection method, as its influence on analyzing microbial community composition (Zhou et al., 2011). To be conservative, it is ideal to adopt a single DNA extraction method for increasing the comparability of different studies. Our results indicate that we can improve the community comparisons between studies that employed different extraction methods with an exception of RT and CRT for their large contributions to community differences among different DNA extraction kits.

Consistency of generating OTU profiles is highly desirable in order to facilitate assessments and comparisons of plankton species diversity and distribution across space and time. Reproducibility is a crucial issue for the study of microbial community ecology. Sample processing steps, from sampling to downstream data analysis, can introduce some biases; such biases can skew data sets by introducing changes in the relative abundances observed, and they can affect the variation among replicates (Zhou et al., 2011; Costea et al., 2017; Pollock et al., 2018; Velásquez-Mejía et al., 2018). The differences in the replicates for each DNA extraction kit are likely the result of DNA extraction and sequencing depth, because other sample process steps should influence all our replicate samples in a similar way (Velásquez-Mejía et al., 2018). Similarly, Zhou et al. (2011) found a reliable diversity comparison across different samples by removing less-representative OTUs for bacteria (e.g., OTUs present only in 1 or 2 of the 14 tag sequence data sets). Our result supported this finding and further demonstrated that the taxa in different relative abundance showed distinct patterns of reproducibility. This could be explained by the high OTUs overlap between replicate samples when low-abundance OTUs were removed from the dataset (Figure 5). In addition, sequencing depth should also be considered, because the percentage of shared OTUs increased as the sequencing depth increase (Liu et al., 2017). However, sequencing depth only exhibited a significant and minor influence on the results of QD and QQ for bacteria, with no significant influence identified for other DNA extraction methods (Supplementary Figure S6). There are also some limitations in this research that need for further study. For example, the technical replicates should be increased to better estimate the background noise level (Zhou et al., 2011). Sample handling environment, and bioinformatics should also be considered for their important influence on the community composition (Costea et al., 2017; Sinha et al., 2017).

## Conclusion

In conclusion, DNA extraction methods had an influence on the results of downstream microbial community analyses, including relative abundances of specific community members for both bacteria and eukaryotes at OTU and higher taxonomic levels. The rare plankton subcommunities are far more affected by DNA extraction kits than the abundant plankton. Every extraction kit was effective, though each showed its own strengths and weaknesses in observing special taxonomic community profiles. MPF produced higher α diversity for bacteria and high quality and yield of DNA, therefore it is the most suitable DNA methods for bacterial plankton diversity study. While QD and QQ methods could produce results that are easier to replicate for bacteria and eukaryotes, respectively. Moreover, our results highlight that the comparability between studies that employed different extraction methods can be improved by removing low-abundance OTUs for their larger contribution to community variation. There is no doubt that the efforts to assess bias within labs as proposed here, and guidelines for best practices across labs, will facilitate comparative studies to characterize microbial communities from multiple sampling surveys and various environments.

## Data Availability

The datasets generated for this study can be found in National Center for Biotechnology Information (NCBI) Sequence Read Archive (SRA) database, BioProject number PRJNA474064 and the accession number SRP149868.

## Author Contributions

JY designed the research. ML and YX performed the experiments. ML and JY analyzed the data and wrote the manuscript. All authors have significantly contributed to the writing of the manuscript.

## Conflict of Interest Statement

The authors declare that the research was conducted in the absence of any commercial or financial relationships that could be construed as a potential conflict of interest.
