# Supplementary material for: Rare Plankton Subcommunities Are Far More Affected by DNA Extraction Kits Than Abundant Plankton
Source: Front Microbiol. 2019 Mar 11;10:454. doi: 10.3389/fmicb.2019.00454 (PMC6423910; doi:10.3389/fmicb.2019.00454)
Supplement: Supplementary file 1 [file Table_1.doc]

**Journal: Frontiers in Microbiology**

*Supplementary information of the article:*

**Rare plankton subcommunities are far more affected by DNA extraction kits than abundant plankton**

Min Liu^1, 2^, Yuanyuan Xue^1, 2^ and Jun Yang^1^, *

^1^ *Aquatic EcoHealth Group, Key Laboratory of Urban Environment and Health, Institute of Urban Environment, Chinese Academy of Sciences, Xiamen 361021, China*

^2^ *University of Chinese Academy of Sciences, Beijing 100049, China*

***Correspondence**:

*E-mail address:* [jyang@iue.ac.cn](mailto:jyang@iue.ac.cn) (Jun Yang); Tel. / Fax: +86-592-6190775.

**This supplementary information contains:**

- 16 Pages
- 7 Figures
- 2 Tables
- 17 References

**
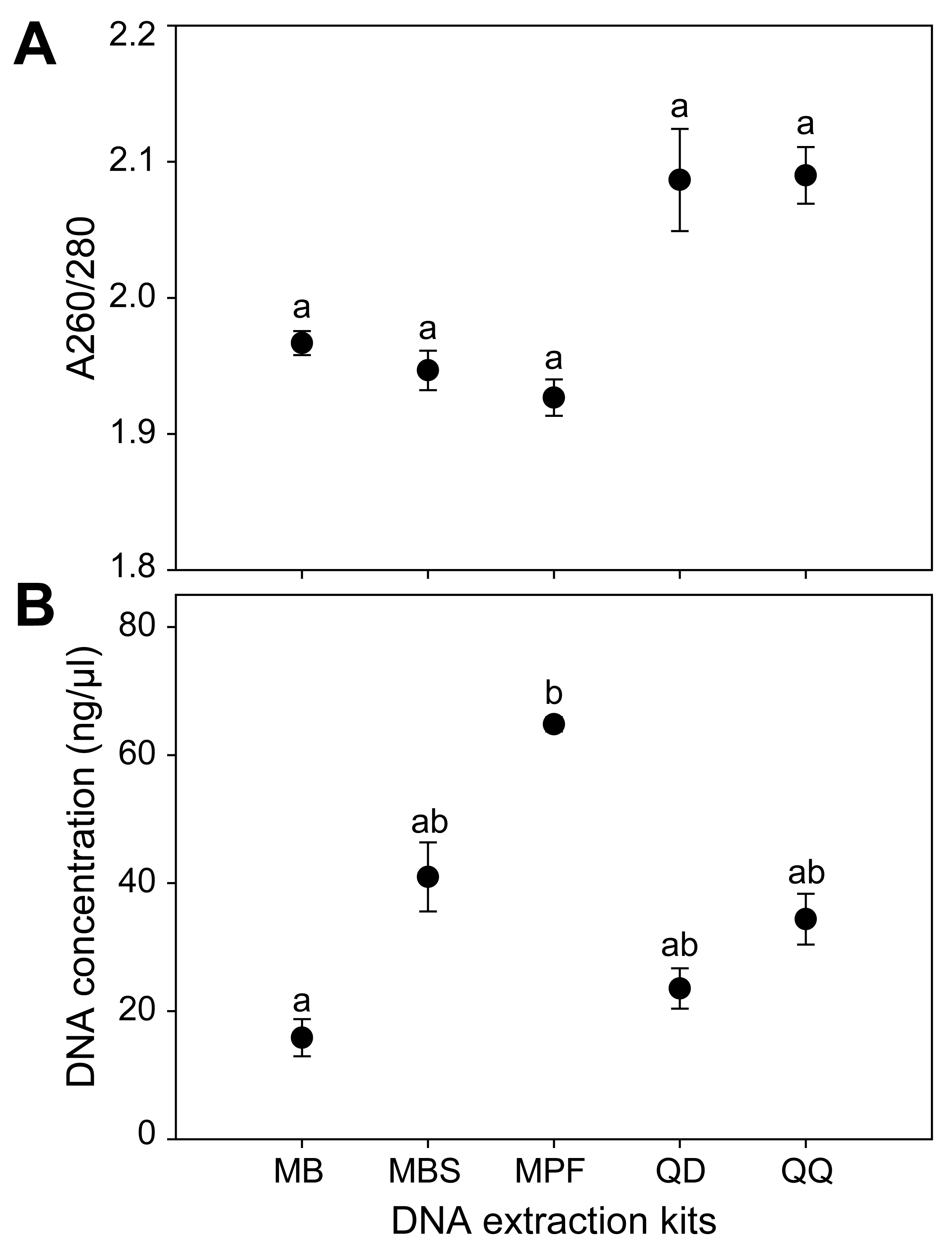
**

**FIGURE S1 |** Effect of DNA extraction kits on DNA quality **(A)** and quantity **(B)**. Data are means ± standard error (n = 3). Significant difference (*P* < 0.05) is indicated by different letters, and the significance is calculated by nonparametric Kruskal-Wallis test.

**
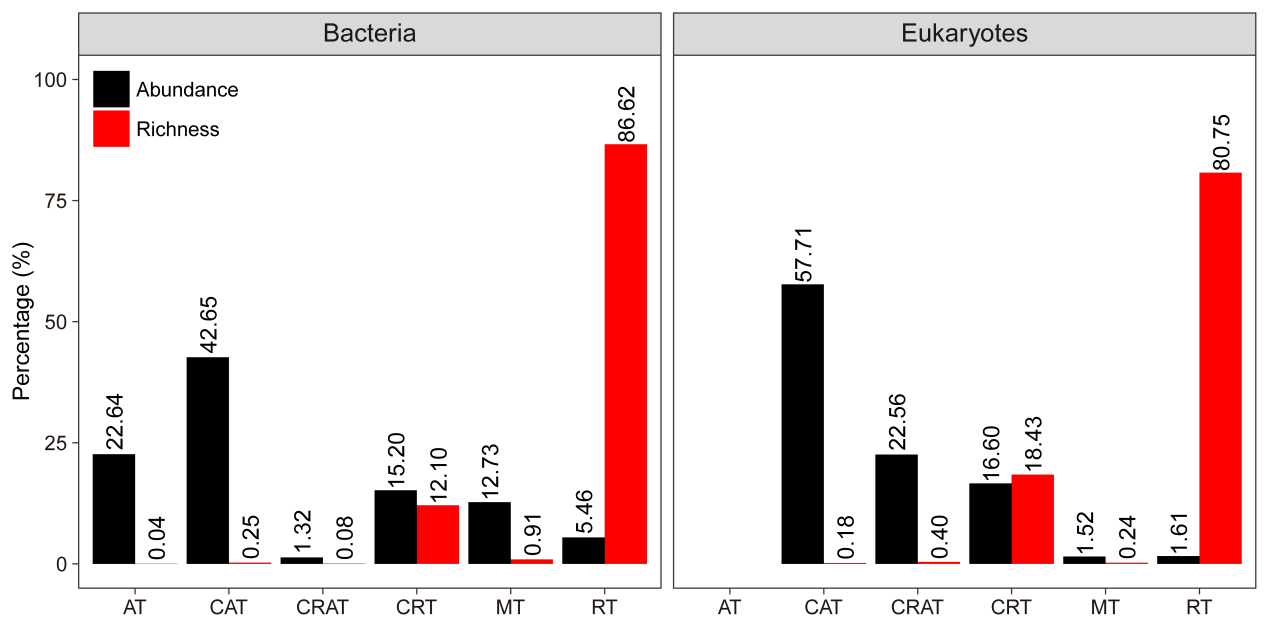
FIGURE S2 |** Contribution of each microbial taxa category to bacterial and eukaryotic plankton community. Always abundant taxa (AT) were defined as the OTUs with a relative abundance always ≥ 1% in 15 replicate samples. Note that no always abundant taxa (AT) was identified for eukaryotic plankton in this study. Conditionally abundant taxa (CAT) were defined as the OTUs with a relative abundance greater than 0.01% in all replicate samples and ≥ 1% in some samples but never rare (< 0.01%). Conditionally rare and abundant taxa (CRAT) were defined as the OTUs with a relative abundance varying from rare (< 0.01%) to abundant (≥ 1%). Moderate taxa (MT) were defined as the OTUs with relative abundance between 0.01% and 1% in all replicate samples. Conditionally rare taxa (CRT) were defined as the OTUs with a relative abundance < 0.01% in some replicate samples but never ≥ 1% in all replicate samples. Always rare taxa (RT) were defined as the OTUs with a relative abundance always < 0.01% in all replicate samples.

**
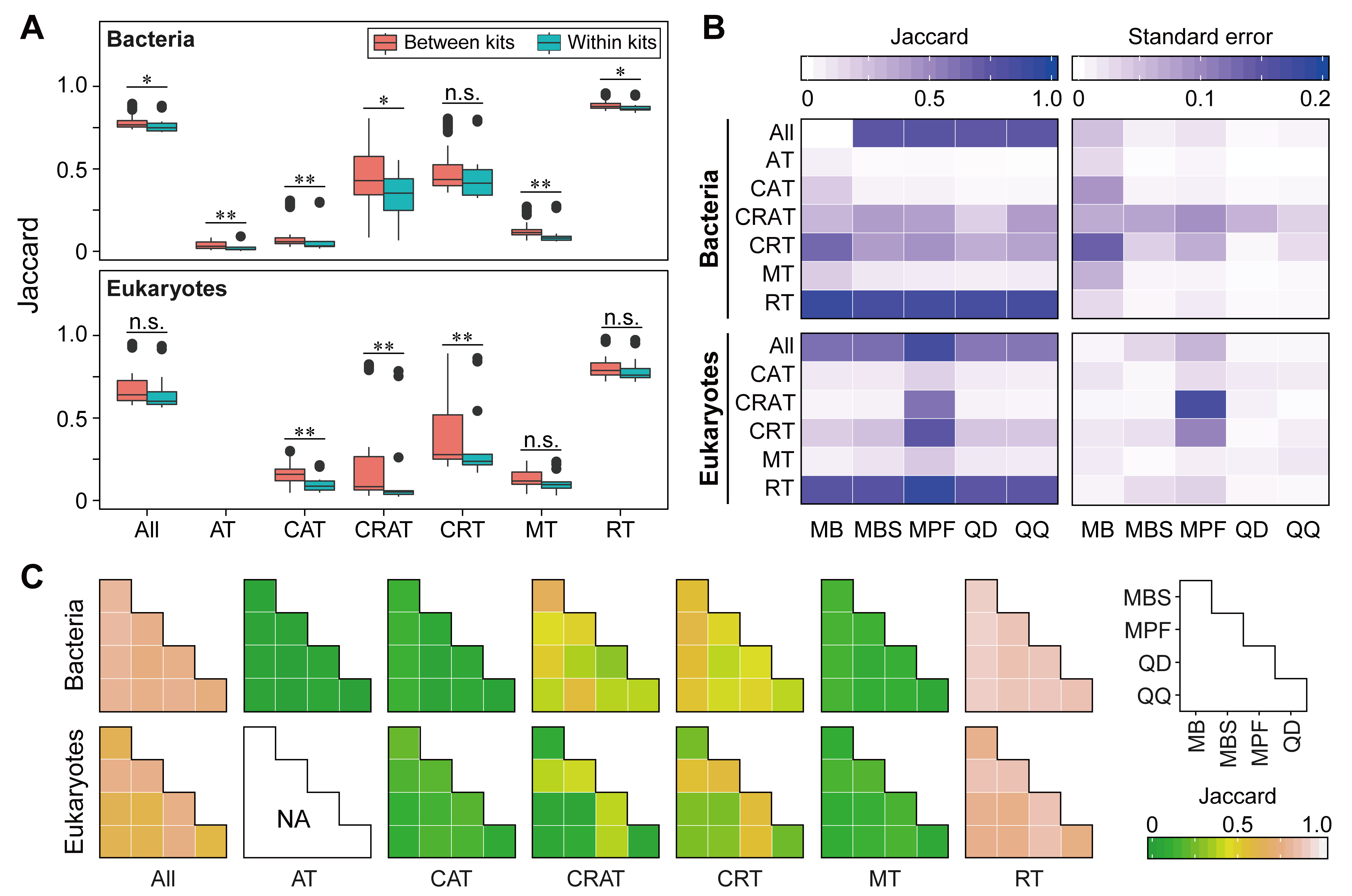
**

**FIGURE S3 |** The effect of DNA extraction kits on community composition of both bacterial and eukaryotic plankton based on Jaccard distance. **(A)** The overview of pairwise Jaccard distance of bacterial and eukaryotic plankton communities based on five different DNA extraction kits, respectively. Statistical analysis is nonparametric Mann-Whitney *U* test. * *P* < 0.05, ** *P* < 0.01, n.s. *P* > 0.05. **(B)** Variation in the community composition of six OTUs categories produced by three replicates within the DNA extractions kits. The data were expressed as mean dissimilarity (left) and its standard error (right), with a more dark blue indicating a more dissimilar or larger variation. **(C)** Pairwise Jaccard distance of bacterial and eukaryotic plankton communities between different DNA extraction kits. Note that no always abundant taxa (AT) was identified for eukaryotic plankton in this study. AT, always abundant taxa; CAT, conditionally abundant taxa; CRAT, conditionally rare and abundant taxa; CRT, conditionally rare taxa; MT, moderate taxa; RT, always rare taxa.

**
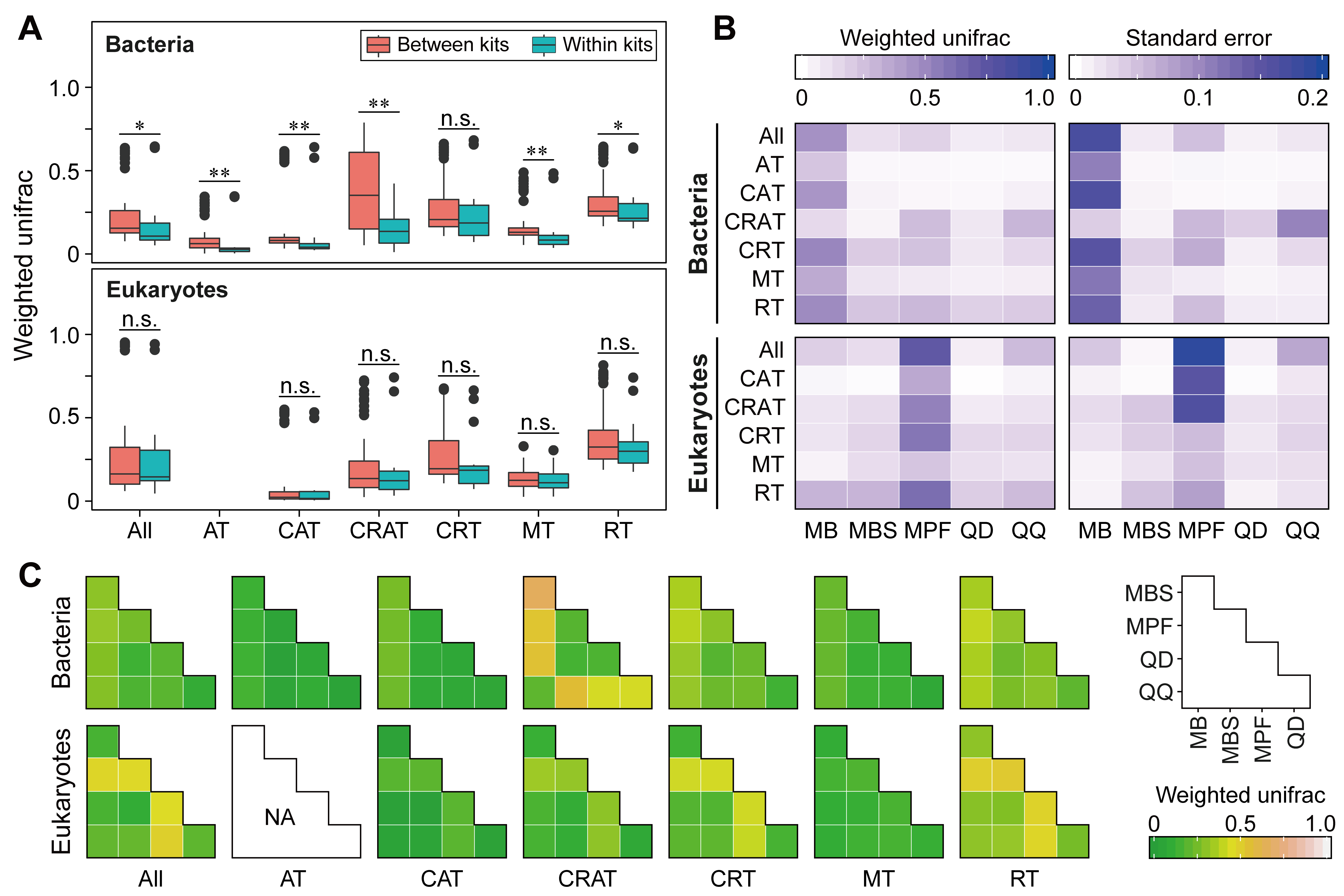
**

**FIGURE S4 |** The effect of DNA extraction kits on community composition of both bacterial and eukaryotic plankton based on weighted UniFrac distance. **(A)** The overview of pairwise weighted UniFrac distance of bacterial and eukaryotic plankton communities based on five different DNA extraction kits, respectively. Statistical analysis is nonparametric Mann-Whitney *U* test. * *P* < 0.05, ** *P* < 0.01, n.s. *P* > 0.05. **(B)** Variation in the community composition of six OTUs categories produced by three replicates within the DNA extractions kits. The data were expressed as mean dissimilarity (left) and its standard error (right), with a more dark blue indicating a more dissimilar or larger variation. **(C)** Pairwise weighted UniFrac distance of bacterial and eukaryotic plankton communities between different DNA extraction kits. Note that no always abundant taxa (AT) was identified for eukaryotic plankton in this study. AT, always abundant taxa; CAT, conditionally abundant taxa; CRAT, conditionally rare and abundant taxa; CRT, conditionally rare taxa; MT, moderate taxa; RT, always rare taxa.

**
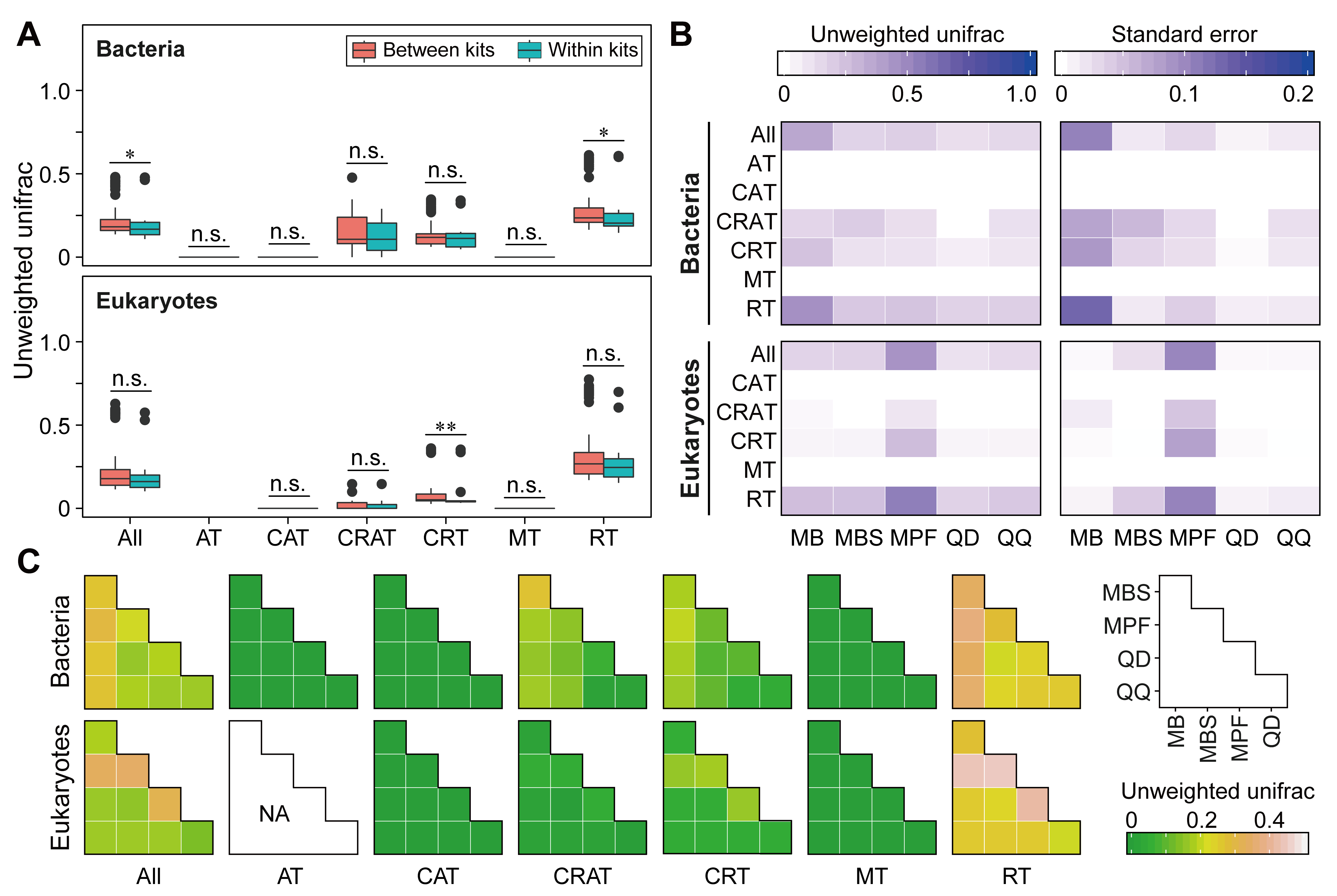
**

**FIGURE S5 |** The effect of DNA extraction kits on community composition of both bacterial and eukaryotic plankton based on unweighted UniFrac distance. **(A)** The overview of pairwise unweighted UniFrac distance of bacterial and eukaryotic plankton communities based on five different DNA extraction kits, respectively. Statistical analysis is nonparametric Mann-Whitney *U* test. * *P* < 0.05, ** *P* < 0.01, n.s. *P* > 0.05. **(B)** Variation in the community composition of six OTUs categories produced by three replicates within the DNA extractions kits. The data were expressed as mean dissimilarity (left) and its standard error (right), with a more dark blue indicating a more dissimilar or larger variation. **(C)** Pairwise unweighted UniFrac distance of bacterial and eukaryotic plankton communities between different DNA extraction kits. Note that no always abundant taxa (AT) was identified for eukaryotic plankton in this study. AT, always abundant taxa; CAT, conditionally abundant taxa; CRAT, conditionally rare and abundant taxa; CRT, conditionally rare taxa; MT, moderate taxa; RT, always rare taxa.


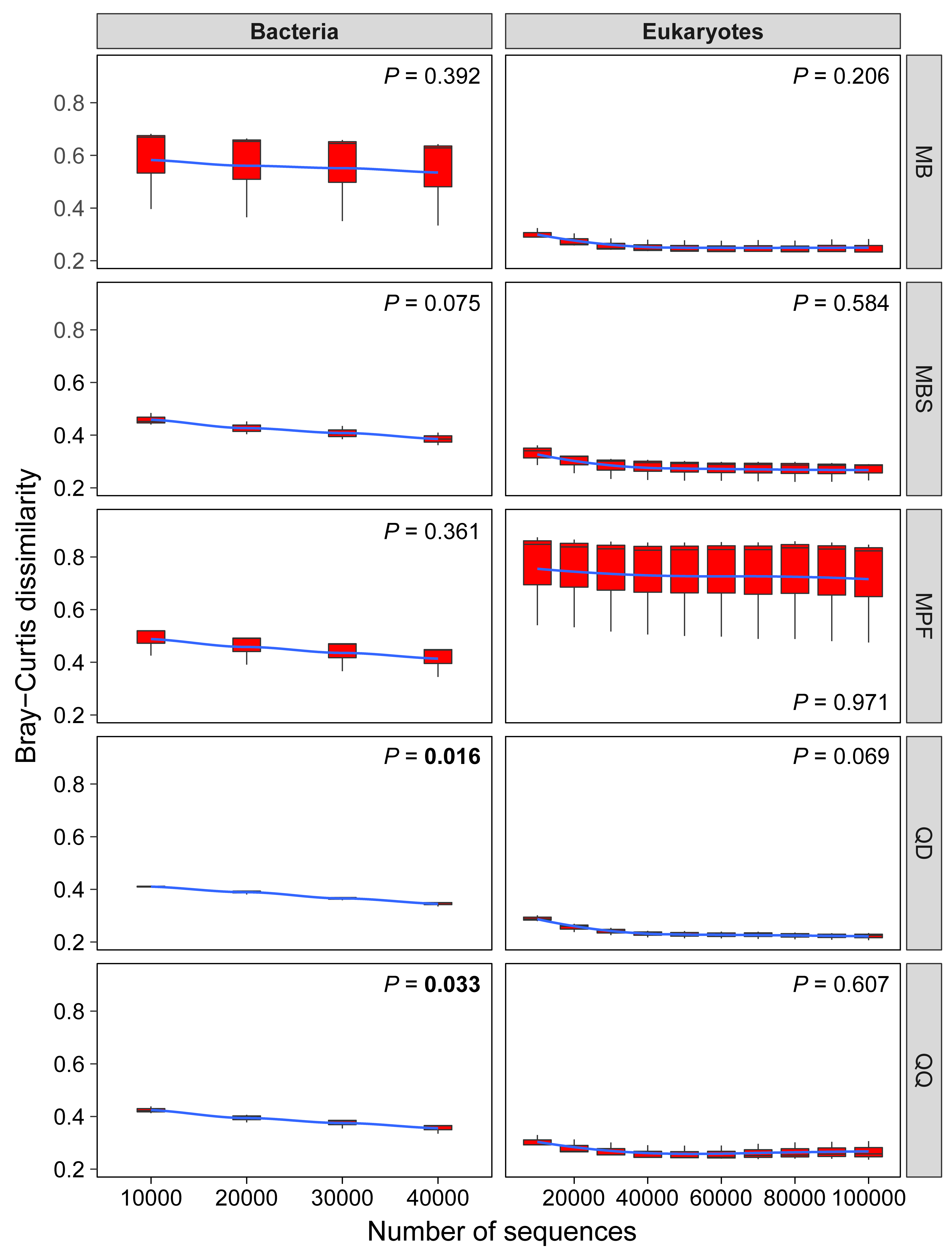


**FIGURE S6 |** Effects of data pre-processing on resolving the differences of microbial communities. The overview of pairwise Bray-Curtis dissimilarity of bacterial and eukaryotic plankton communities produced by three replicates within the five DNA extraction kits at different sequencing depths. Statistical analysis is nonparametric Kruskal-Wallis test, and *P* values are indicated.

**
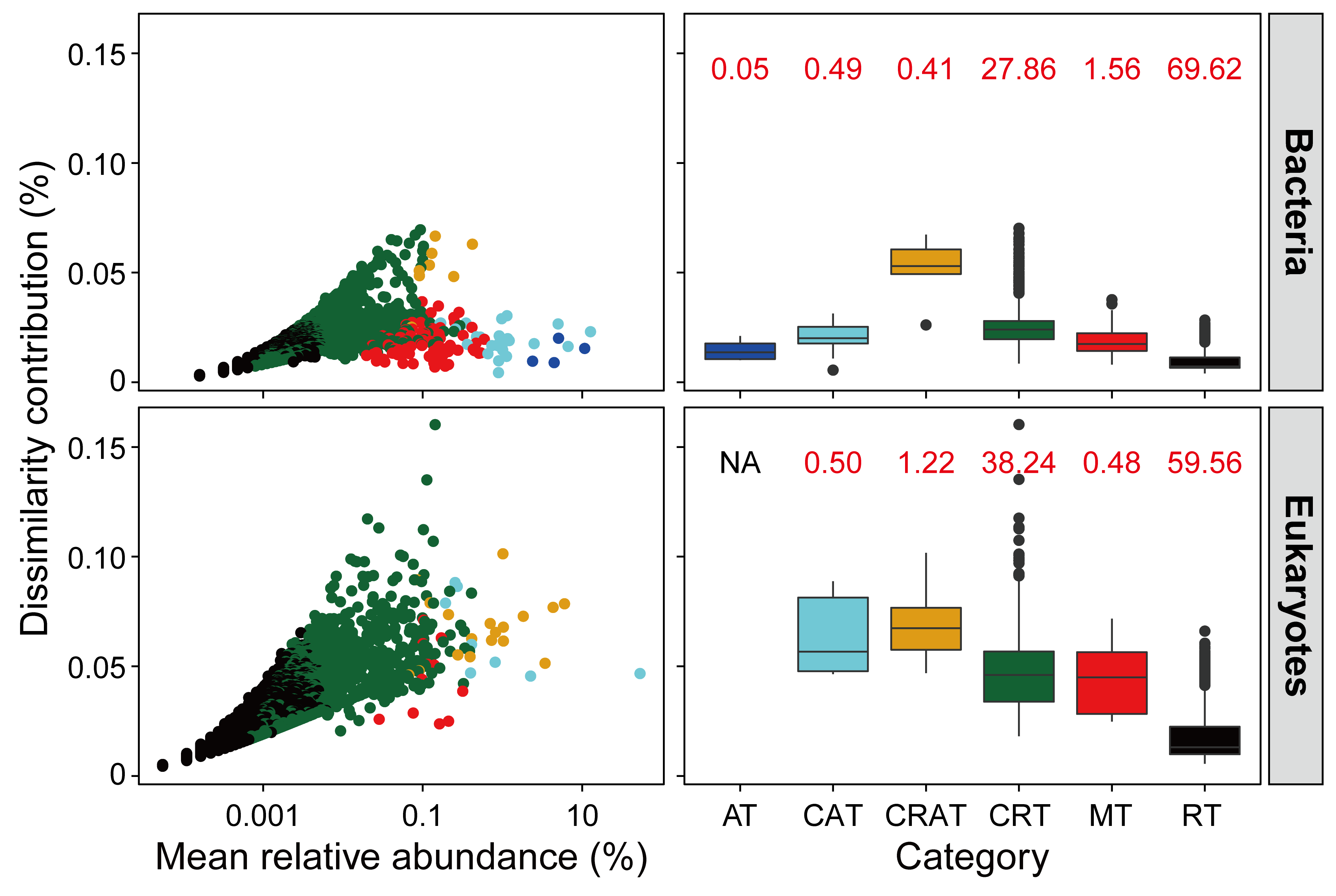
**

**FIGURE S7 |** Community dissimilarity contribution of all OTUs for bacteria and eukaryotes based on SIMPER analysis. Numbers in red of each category represent the total contribution (%) to community dissimilarity. Note that no always abundant taxa (AT) was identified for eukaryotic plankton in this study. AT, always abundant taxa; CAT, conditionally abundant taxa; CRAT, conditionally rare and abundant taxa; CRT, conditionally rare taxa; MT, moderate taxa; RT, always rare taxa.

**Table S1 |** Previous literatures analyzing the influence of DNA extraction on bacterial and eukaryotic communities.

| Sample origin | Taxa | Community | DNA extraction method | References |
| --- | --- | --- | --- | --- |
| Carp | Fish | Detection, quantification | MoBio PowerSoil DNA Isolation Kit,  MoBio PowerWater DNA Isolation Kit,  MP Biomedicals FastDNA SPIN Kit,  MP Biomedicals FastDNA SPIN Kit for Soil,  Qiagen DNeasy Blood and Tissue Kit,  Qiagen QIAamp DNA Stool Mini Kit | Eichmiller et al., 2016 |
| Clinical samples | Fungi | Quantification | MasterPure yeast DNA purification kit, UltraClean soil DNA isolation kit, FastDNA kit, MasterPure plant leaf DNA purification kit, yeast cell lysis preparation kit plus GNOME kit, SoilMaster DNA extraction kit, QIAamp DNA stool mini kit | Fredricks et al., 2005 |
| Cow and sheep  rumen | Bacteria, archaea | Entire community | 15 DNA extraction protocols | Henderson et al., 2013 |
| Cultured *Salmonella*  *bongori strain NCTC-12419*, human | Bacteria | Entire community, low-biomass microbiota | FastDNA Spin Kit For Soil, UltraClean Microbial DNA Isolation Kit,  QIAmp DNA Stool Mini Kit,  PSP Spin Stool DNA Plus kit | Salter et al., 2014 |
| Faeces | Bacteria | Entire community | 21 representative DNA extraction protocols | Costes et al., 2017 |
|  |  |  | PowerSoil DNA Isolation Kit, QIAamp DNA Stool Mini Kit | Velásquez-Mejía et al., 2018 |

**Table S1 | Continued**

| Soil | Protist | Targeted species | Power Lyzer™ PowerSoil® DNA Isolation Kit, ISO standard methods (ISOm) modified, GnS-GII protocol | Santos et al., 2015 |
| --- | --- | --- | --- | --- |
|  |  |  | Power Lyzer™ PowerSoil® DNA Isolation Kit, ISO standard methods (ISOm) modified, GnS-GII protocol | Santos et al., 2017 |
| Water | Macroinvertebrate, eubacteria, eukaryotes | Targeted species,  entire community | Qiagen’s DNeasy Blood & Tissue Kit,  MO BIO’s PowerWater DNA Isolation Kit,  Modified phenol–chloroform–isoamyl extraction, | Deiner et al., 2015 |
|  | Bacteria, Archaea, phytoplankton, vertebrates | Entire community | DNeasy Blood and Tissue kit, MoBio PowerWater DNA Isolation kit,  Standard phenol/chloroform methods | Djurhuus et al., 2017 |
|  | Phytoplankton |  | QiagenDNeasy® Plant Mini kit,  QiagenDNeasy® Blood and Tissue kit,  MoBioUltraCleanTM Soil DNA Isolation kit | Eland et al., 2012 |
|  |  |  | DNeasy Plant Mini Kit, Power Water DNA Isolation Kit, Power BiofilmDNA Isolation Kit | Mäki et al., 2017 |
|  | Bacteria |  | QIAamp DNA Mini Kit, QIAamp DNA Stool Mini Kit, MO BIO Power Water Kit, MO BIO Power Soil DNA Isolation Kit | Walden et al., 2017 |

**Table S1 | Continued**

| Soil, water, biofilm, sediment, leaf litter  and kick-net | Bacteria, fungi, animals, plant, fish | Entire community | Qiagen DNeasy Blood & Tissue Kit, Qiagen DNeasy PowerSoil Kit, MoBio PowerWater DNA Isolation Kit, Qiagen DNeasy Plant Mini Kit, Qiagen DNeasy Plant Mini Kit, ZymoBIOMICS DNA Miniprep Kit | Hermans et al., 2018 |
| --- | --- | --- | --- | --- |
| Human, pig, sewage | Bacteria | Entire community | InnuPure C16 from Analytic Jena AG,  MagNA Pure LC DNA isolation kit III from Roche,  Easy-DNA genomic DNA (gDNA) purification kit from Invitrogen,  MP FastDNA Spin kit from MP Biomedicals,  PowerSoil DNA isolation kit from MoBio,  QIAamp DNA stool minikit from Qiagen,  QIAamp DNA stool minikit plus bead beating from Qiagen | Knudsen et al., 2016 |
| Activated sludge, broad range, plants, soil, liquid culture | Bacteria | Entire community | 10 different DNA extraction procedures | Kuhn et al., 2017 |
| Human stool, chemostats, artificial microbial communities | Bacteria | Entire community | PowerMax Soil DNA Isolation Kit,  MO-BIO PowerSoil kit,  MO-BIO PowerMag kit,  MO-BIO PowerSoil, Qiagen QIAamp, In-house protocol, and so on | Sinha et al., 2017 |

**Table S2 |** Indicator OTUs for the five DNA extraction kits. Only OTUs with *P*-value < 0.05 and those with phylotypes with indicator values > 50 were considered as valid indicator OTUs (see Materials and Methods).

| OTU ID | Category | *P* | IndVal | Taxonomy |
| --- | --- | --- | --- | --- |
| **Bacteria** | | | | |
| **MB (n = 1)** | | | | |
| OTU_159 | CRT | 0.004** | 76.21 | Bacteria |
| **MPF(**n = 6) | | | | |
| OTU_103 | CRT | 0.018* | 50.67 | Acidobacteria; Acidobacteria; Subgroup_4; 11-24 |
| OTU_112 | CRT | 0.004** | 51.18 | Actinobacteria; Acidimicrobiia; Acidimicrobiales |
| OTU_208 | CRT | 0.031* | 90.67 | Actinobacteria; Actinobacteria; Frankiales; Sporichthyaceae; hgcI_clade |
| OTU_385 | CRT | 0.009** | 62.44 | Proteobacteria; Alphaproteobacteria; Rhodospirillales; Rhodospirillales_Incertae_Sedis; *Reyranella* |
| OTU_480 | CRT | 0.004** | 50.30 | Proteobacteria; Alphaproteobacteria; Rhizobiales |
| OTU_714 | CRT | 0.004** | 63.27 | Actinobacteria; Actinobacteria; Frankiales; Sporichthyaceae; hgcI_clade |
| **QQ (n = 2)** | | | | |
| OTU_334 | CRT | 0.004** | 51.75 | TM6 |
| OTU_458 | CRT | 0.004** | 50.33 | Cyanobacteria; Cyanobacteria; SubsectionI; FamilyI |
| **Eukaryotes** | | | | |
| **MB (n = 4)** | | | | |
| OTU_26 | CAT | 0.031* | 51.15 | Archaeplastida; Chlorophyta; Chlorophyceae; Chlorophyceae_X; Sphaeropleales; *Monoraphidium; Monoraphidium_dybowskii* |
| OTU_27 | CAT | 0.018* | 51.50 | Archaeplastida; Chlorophyta; Chlorophyceae; Chlorophyceae_X; Sphaeropleales; *Tetranephris; Tetranephris_brasiliensis* |
| OTU_84 | CRT | 0.018* | 51.43 | Archaeplastida; Chlorophyta; Chlorophyceae; Chlorophyceae_X; Sphaeropleales; *Characium; Characium_californicum* |
| OTU_1129 | RT | 0.004** | 55.56 | Archaeplastida; Chlorophyta; Chlorophyceae; Chlorophyceae_X; Sphaeropleales; *Monoraphidium; Monoraphidium_*sp. |

**Table S2 | Continued**

| OTU ID | Category | *P* | | IndVal | Taxonomy |
| --- | --- | --- | --- | --- | --- |
| **MPF (n = 13)** | | | | | |
| OTU_33 | CAT | 0.009** | | 57.75 | Archaeplastida; Chlorophyta; Chlorophyceae; Chlorophyceae_X; Sphaeropleales; *Pseudopediastrum; Pseudopediastrum_boryanum* |
| OTU_46 | CRAT | 0.004** | | 83.52 | Archaeplastida; Chlorophyta; Trebouxiophyceae; Chlorellales; Chlorellales_X; *Oocystella;Oocystella_oogama* |
| OTU_116 | CRT | 0.031* | | 93.17 | Archaeplastida; Chlorophyta; Chlorophyceae; Chlorophyceae_X; Sphaeropleales; Desmodesmus; *Desmodesmus_communis* |
| OTU_131 | CRT | 0.004** | | 82.14 | Archaeplastida; Chlorophyta; Chlorophyta_X; Chlorophyta_XX; Chlorophyta_XXX; *Oocystella;Oocystella_oogama* |
| OTU_134 | CRT | 0.009** | | 93.99 | Archaeplastida; Chlorophyta; Chlorophyceae; Chlorophyceae_X; Sphaeropleales; *Scenedesmus; Scenedesmus_armatus* |
| OTU_136 | CRT | 0.009** | | 74.83 | Archaeplastida; Chlorophyta; Trebouxiophyceae; Chlorellales; Chlorellales_X; *Oocystis; Oocystis_parva* |
| OTU_236 | CRT | 0.048* | | 51.18 | Archaeplastida; Chlorophyta; Trebouxiophyceae; Chlorellales; Chlorellales_X; Franceia; Franceia_amphitricha |
| OTU_242 | CRT | 0.009** | | 89.16 | Archaeplastida; Chlorophyta; Chlorophyceae; Chlorophyceae_X; Sphaeropleales; *Desmodesmus; Desmodesmus_communis* |
| OTU_296 | CRT | 0.031* | | 82.70 | Opisthokonta; Mesomycetozoa; Ichthyosporea; Ichthyosphonida; Ichthyophonidae_Freshwater; Ichthyophonidae_Freshwater_X; Ichthyophonidae_Freshwater_X_sp. |
| OTU_345 | CRT | 0.031* | | 58.43 | Archaeplastida; Chlorophyta; Trebouxiophyceae; Chlorellales; Chlorellales_X; *Chlorella;Chlorella_vulgaris* |
| OTU_440 | CRT | 0.009** | | 86.96 | Excavata; Discoba; Euglenozoa; Euglenida; Euglenales; *Trachelomonas; Trachelomonas_volvocinopsis* |
| OTU_711 | CRT | 0.018* | | 60.71 | Archaeplastida; Chlorophyta; Trebouxiophyceae; Chlorellales; Chlorellales_X; *Hindakia; Hindakia_fallax* |
| OTU_787 | CRT | 0.004** | | 70.59 | Stramenopiles; Ochrophyta; Chrysophyceae-Synurophyceae; Chrysophyceae-Synurophyceae_X; Clade-F; Clade-F_X; Clade-F_X_sp. |
| **QQ (n = 7)** | | | | | |
| OTU_65 | CRT | 0.004** | 60.83 | | Archaeplastida; Chlorophyta; Chlorophyceae; Chlorophyceae_X; Sphaeropleales;*Sphaeropleales; Sphaeropleales*_sp. |
| OTU_71 | CRT | 0.004** | 55.54 | | Archaeplastida; Chlorophyta; Chlorophyceae;Chlorophyceae_X; Sphaeropleales; *Pseudopediastrum; Pseudopediastrum_boryanum* |
| OTU_208 | CRT | 0.004** | 61.76 | | Archaeplastida; Chlorophyta; Chlorophyceae; Chlorophyceae_X; Sphaeropleales; *Follicularia; Follicularia_texensis* |
| OTU_227 | CRT | 0.004** | 55.89 | | Archaeplastida; Chlorophyta; Chlorophyceae; Chlorophyceae_X; Sphaeropleales; *Radiococcus; Radiococcus*_sp. |
| OTU_368 | CRT | 0.004** | 53.98 | | Archaeplastida; Chlorophyta; Chlorophyceae; Chlorophyceae_X; CW-Chlamydomonadales; *Tetracystis; Tetracystis_sarcinalis* |

**Table S2 | Continued**

| OTU ID | Category | *P* | IndVal | Taxonomy |
| --- | --- | --- | --- | --- |
| OTU_409 | CRT | 0.004** | 57.75 | Stramenopiles; Ochrophyta; Eustigmatophyceae; Eustigmatophyceae_X; Eustigmatophyceae_XX; *Nannochloropsis; Nannochloropsis_granulata* |
| OTU_699 | RT | 0.018* | 50.41 | Archaeplastida; Chlorophyta; Chlorophyceae; Chlorophyceae_X; Sphaeropleales; *Follicularia; Follicularia_texensis* |

**P* < 0.05, ***P* < 0.01.

Note that for bacteria and eukaryotes, no indicator was found for both MBS and QD kits.

CAT, conditionally abundant taxa; CRAT, conditionally rare and abundant taxa; CRT, conditionally rare taxa; RT, always rare taxa.

**References**

Costea, P. I., Zeller, G., Sunagawa, S., Pelletier, E., Alberti, A., Levenez, F., et al. (2017). Towards standards for human fecal sample processing in metagenomic studies. *Nat. Biotechnol.* 35, 1069–1076. doi: 10.1038/nbt.3960

Deiner, K., Walser, J. C., Mächler, E., and Altermatt, F. (2015). Choice of capture and extraction methods affect detection of freshwater biodiversity from environmental DNA. *Biol. Conserv.* 183, 53–63. doi: 10.1016/j.biocon.2014.11.018

Djurhuus, A., Port, J., Closek, C. J., Yamahara, K. M., Romero-Maraccini, O., Walz, K. R., et al. (2017). Evaluation of filtration and DNA extraction methods for environmental DNA biodiversity assessments across multiple trophic levels. *Front. Mar. Sci*. 4:314. doi: 10.3389/fmars.2017.00314

Eichmiller, J. J., Miller, L. M., and Sorensen, P. W. (2016). Optimizing techniques to capture and extract environmental DNA for detection and quantification of fish. *Mol. Ecol. Resour*. 16, 56–68. doi: 10.1111/1755-0998.12421

Eland, L. E., Davenport, R., and Mota, C. R. (2012). Evaluation of DNA extraction methods for freshwater eukaryotic microalgae. *Water Res.* 46, 5355–5364. doi: 10.1016/j.watres.2012.07.023

Fredricks, D. N., Smith, C., and Meier, A. (2005). Comparison of six DNA extraction methods for recovery of fungal DNA as assessed by quantitative PCR. *J. Clin. Microbiol.* 43, 5122–5128. doi: 10.1128/JCM.43.10.5122-5128.2005

Henderson, G., Cox, F., Kittelmann, S., Vahideh, H. M., Zethof, M., Noel, S. J., et al. (2013). Effect of DNA extraction methods and sampling techniques on the apparent structure of cow and sheep rumen microbial communities. *PLoS One* 8:e74787. doi: 10.1371/journal.pone.0074787

Hermans, S. M., Buckley, H. L., and Lear, G. (2018). Optimal extraction methods for the simultaneous analysis of DNA from diverse organisms and sample types. *Mol. Ecol. Resour.* 18, 557–569. doi: 10.1111/1755-0998.12762

Knudsen, B. E., Lasse, B., Munk, P., Oksana, L., Priemé Anders, Aarestrup, F. M., et al. (2016). Impact of sample type and DNA isolation procedure on genomic inference of microbiome composition. *mSystems* 1, e00095–e00016. doi: 10.1128/mSystems.00095-16

Kuhn, R., Böllmann, J., Krahl, K., Bryant, I. M., and Martienssen, M. (2017). Comparison of ten different DNA extraction procedures with respect to their suitability for environmental samples. *J. Microbiol. Meth.* 143, 78–86. doi: 10.1016/j.mimet.2017.10.007

Mäki, A., Salmi, P., Mikkonen, A., Kremp, A., and Tiirola, M. (2017). Sample preservation, DNA or RNA extraction and data analysis for high-throughput phytoplankton community sequencing. *Front. Microbiol*. 8:1848. doi: 10.3389/fmicb.2017.01848

Salter, S. J., Cox, M. J., Turek, E. M., Calus, S. T., Cookson, W. O., Moffatt, M. F., et al. (2014). Reagent and laboratory contamination can critically impact sequence-based microbiome analyses. *BMC Biol*. 12:87. <http://dx.doi.org/10.1186/s12915-014-0087-z>

Santos, S. S., Nielsen, T. K., Hansen, L. H., and Winding, A. (2015). Comparison of three DNA extraction methods for recovery of soil protist DNA. *J. Microbiol. Meth.* 115, 13–19. doi: 10.1016/j.mimet.2015.05.011

Santos, S. S., Nunes, I., Nielsen, T. K., Jacquiod, S., Hansen, L. H., and Winding, A. (2017). Soil DNA extraction procedure influences protist 18S rRNA gene community profiling outcome.

*Protist* 168, 283–293. doi: 10.1016/j.protis.2017.03.002

Sinha, R., Abu-Ali, G., Vogtmann, E., Fodor, A. A., Ren, B., Amir, A., et al. (2017). Assessment of variation in microbial community amplicon sequencing by the microbiome quality control (MBQC) project consortium. *Nat. Biotechnol.* 35, 1077–1086. doi: 10.1038/nbt.3981

Velásquez-Mejía, E. P., de la Cuesta-Zuluaga, J., and Escobar, J. S. (2018). Impact of DNA extraction, sample dilution, and reagent contamination on 16S rRNA gene sequencing of human feces. *Appl. Microbiol. Biot.* 102, 403–411. doi: 10.1007/s00253-017-8583-z

Walden, C., Carbonero, F., and Zhang, W. (2017). Assessing impacts of DNA extraction methods on next generation sequencing of water and wastewater samples. *J. Microbiol. Meth.* 141, 10–16. doi: 10.1016/j.mimet.2017.07.007
